# 23-Valent Pneumococcal Polysaccharide Vaccination Does Not Prevent Community-Acquired Pneumonia Hospitalizations Due to Vaccine-Type *Streptococcus pneumoniae*

**DOI:** 10.3390/microorganisms10030560

**Published:** 2022-03-04

**Authors:** Thomas Chandler, Stephen Furmanek, Ruth Carrico, Dawn Balcom, Forest Arnold, Julio Ramirez

**Affiliations:** 1 Norton Infectious Diseases Institute, 601 South Floyd Street, Suite 603, Louisville, KY 40202, USA; stephen.furmanek@nortonhealthcare.org (S.F.); ruth.carrico@nortonhealthcare.org (R.C.); julio.ramirez@nortonhealthcare.org (J.R.); 2 Division of Infectious Diseases, University of Louisville, Louisville, KY 40202, USA; dawn.balcom@louisville.edu (D.B.); f.arnold@louisville.edu (F.A.)

**Keywords:** community-acquired pneumonia, pneumococcal pneumonia, pneumococcal vaccine, polysaccharide vaccine

## Abstract

Controversy exists regarding the clinical effectiveness of the 23-valent pneumococcal polysaccharide vaccine (PPSV23) for the prevention of serotype-specific community-acquired pneumonia (CAP). The objective of this study was to define the effectiveness of PPSV23 for the prevention of CAP hospitalizations due to vaccine-contained serotypes. This secondary analysis was a nested case–control, test-negative study design of adult patients hospitalized for CAP between 1 June 2014 and 31 March 2017. Cases included patients with CAP due to a *S. pneumoniae* serotype contained in the PPSV23. Urinary antigen detection of the 23 serotypes was performed. In the study, PPSV23 vaccination alone and no other pneumococcal vaccination was the primary exposure of interest. Vaccine effectiveness was calculated as (1-OR) × 100. Adjusted estimates were obtained from a logistic regression model that controlled for confounding variables. A total of 3686 patients were included in the analysis. The PPSV23 vaccination was documented in 608 (16%) patients, and the PPSV23-serotype CAP was detected in 48 (8%) PPSV23-vaccinated patients and in 288 (9%) non-vaccinated patients. Unadjusted vaccine effectiveness for preventing PPSV23-serotype CAP was 17% (95% CI: −13% to 40%). Adjusted estimates for preventing PPSV23-serotype CAP was 14% (95% CI: −17% to 38%). In this study, PPSV23 vaccination offered no protection against PPSV23-serotype CAP hospitalization in adults. This is the first PPSV23 vaccine effectiveness study from United States that utilized a urinary antigen detection assay as the main method for *S. pneumoniae* serotyping. This study highlights the need for more effective vaccines in the prevention of hospitalization due to *S. pneumoniae* CAP.

## 1. Introduction

In the United States, the 23-valent pneumococcal polysaccharide vaccine (PPSV23) was approved for use in 1983 for the prevention of pneumococcal disease caused by the 23 *Streptococcus pneumoniae* serotypes contained in the vaccine. Current data suggests that PPSV23 vaccination is effective in preventing invasive pneumococcal disease (IPD) in adults [1,2,3,4,5], with an estimated effectiveness of 60% to 70% reported by the United States Centers for Disease Control and Prevention (CDC) [6]. However, there is no consensus regarding PPSV23 vaccination for the prevention of hospitalized CAP due to serotypes contained in the PPSV23. The primary challenge with this type of study has been the identification of vaccine-specific serotypes. New technologies using non-culture-based techniques such as the 13-valent Luminex multiplex urinary antigen detection (UAD) assay have demonstrated high sensitivity and specificity for serotypes covered by the 13-valent pneumococcal conjugated vaccine (PCV13) in adults hospitalized with CAP [7]. More recently, increased serotype coverage available in the UAD-2 assay now facilitates the identification of 23 serotypes in hospitalized adult patients [8]. With the UAD-2 assay, the estimation of PPSV23 vaccine effectiveness (VE) for the prevention of hospitalized vaccine-type infections is now possible. There is a paucity of data concerning the PPSV23 for the prevention of hospitalized CAP due to *S. pneumoniae* serotypes contained in the PPSV23 (PPSV23-serotype CAP). The objective of this study was to define the effectiveness of the PPSV23 for the prevention of hospitalized PPSV23-serotype CAP.

## 2. Materials and Methods

This secondary analysis was a nested case–control, test-negative study design of hospitalized patients from the University of Louisville Pneumonia Study (ULPS) database [8]. The ULPS included all consecutive hospitalized patients with a diagnosis of CAP in the city of Louisville, Kentucky. Patients were eligible for inclusion into the ULPS if they were hospitalized for CAP at any of the nine adult acute-care hospitals in Louisville from 1 June 2014–31 March 2017.

For this secondary analysis, patients were included if they consented to the collection of a urine sample. Patients were excluded if a urine sample was not obtained, had prior PCV13 vaccination, and/or vaccination status was not documented in a patient’s electronic medical record (EMR) or primary insurer.

Patient data were abstracted from hospital EMRs. This included age, sex, race, body mass index, type of residence, medical and social history, physical examination findings, laboratory findings, and severity of illness.

Community-acquired pneumonia (CAP) was defined as meeting the following three criteria: (1) presence of a new pulmonary infiltrate on chest radiograph and/or chest computed tomography scan at the time of hospitalization, defined by a board-certified radiologist’s reading; (2) at least one of the following: (a) new cough or increased cough or sputum production, (b) fever >37.8 °C (100.0 °F) or hypothermia, (c) white blood cell count of >11,000 cells/μL, left shift of >10% band forms/mL, or leukopenia (<4000 cells/μL); and (3) no alternative diagnosis at the time of hospital discharge that justified the presence of criteria 1 and 2.

The identification of serotypes 1, 2, 3, 4, 5, 6B, 7F, 8, 9N, 9V, 10A, 11A, 12F, 14, 15B, 17F, 18C, 19F, 19A, 20, 22F, 23F, and 33F was performed in all eligible patients using the UAD-2 assay [9]. Cases included patients who tested positive on the UAD-2 for any of the 23 serotypes found in the PPSV23 vaccine. Patients who tested negative for all of the 23 serotypes found in the PPSV23 vaccine were defined as test-negative controls.

The PPSV23 vaccination, and no other pneumococcal vaccination, was the primary exposure of interest. Patients were classified as PPSV23-vaccinated or non-vaccinated based on their PPSV23 vaccination history. Patients who received a PPSV23 vaccination within the prior 30 days, or more than five years prior to their CAP hospitalization were considered to be non-vaccinated. Vaccination status was confirmed by EMRs and/or primary health insurer.

Severity of illness was assessed using three different measures and included the following: (1) direct admission to the intensive care unit (ICU) on their first day of hospitalization; (2) Pneumonia Severity Index (PSI) risk classes IV or V [10]; and (3) a CURB-65 score of 4 or 5 [11].

Baseline characteristics were reported between PPSV23-vaccinated and non-vaccinated individuals, and between patients with PPSV23-serotype CAP and non-PPSV23-serotype CAP. Categorical variables were summarized as frequencies and percentages and compared using chi-squared tests of independence. Continuous variables were summarized as median and interquartile ranges (IQR) and compared using Mann–Whitney U-tests.

Vaccine effectiveness (VE) in the overall cohort was calculated as (1-OR) × 100 [12]. Adjusted estimates were obtained from a logistic regression model. Confounding variables included in this model were selected with a priori knowledge of risk factors associated with *S. pneumoniae* infection, underlying comorbidities, and immune status based on current vaccine recommendations [13]. These included age, diabetes, chronic obstructive pulmonary disease (COPD), congestive heart failure, and hyperlipidemia. Subgroup analyses were performed for the following patient groups: patients aged 65 and older, patients directly admitted to the ICU, patients admitted to the ward, patients in PSI risk classes IV–V, patients in PSI risk classes I–III, patients with a CURB-65 score of 4–5, and patients with a CURB-65 score of 0–3. A sensitivity analysis excluding patients who were PPSV23-vaccinated within the prior 30 days, or more than five years prior to their CAP hospitalization was also performed for the primary analysis. All analyses were performed in R version 4.1.1. *p*-values were two-sided with statistical significance set at *p* < 0.05.

This study was approved by the Institutional Review Board (IRB) at the University of Louisville Human Subjects Research Protection Program Office (IRB numbers 11.0613, 12.0470, 13.0408) and by the research offices at each of the nine participating hospitals.

## 3. Results

**Figure 1:**
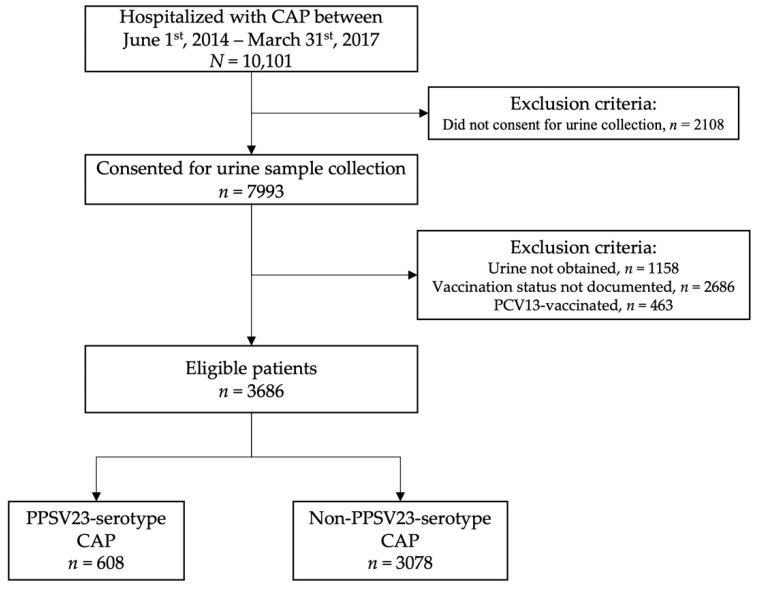
Patient Selection Flowchart.

A total of 10,101 patients hospitalized for CAP were identified from the ULPS database. Consent for urine was obtained in 7993 patients. Of these, both urine samples and proof of vaccination were obtained in 4149 patients. Prior PCV13 vaccination was documented in 463 patients. A total of 3686 patients were eligible and included in analysis. Figure 1 outlines the patient selection process.

Furthermore, PPSV23 vaccination was documented in 608 (16%) patients. Among the PPSV23-vaccinated patients, EMRs were the primary source of vaccination status in 530 (87%) patients, followed by the primary insurer in 78 (13%) patients.

### 3.1. Patient Characteristics by PPSV23-Vaccinated and Non-Vaccinated Patients

Table 1 depicts patient demographics, social history, and medical history, stratified according to PPSV23-vaccinated and non-vaccinated patients. The median age for vaccinated individuals was significantly older (68 vs. 65; *p*-value = 0.001). Vaccinated patients were also more comorbid with significantly higher rates of diabetes (37% vs. 31%; *p*-value = 0.006), COPD (58% vs. 47%; *p*-value < 0.001), renal disease (33% vs. 26%; *p*-value < 0.001), heart failure (36% vs. 28%; *p*-value < 0.001), coronary artery disease (37% vs. 28%; *p*-value < 0.001), hypertension (77% vs. 66%; *p*-value < 0.001), hyperlipidemia (53% vs. 41%; *p*-value < 0.001), prior myocardial infarction (18% vs. 12%; *p*-value < 0.001) and atrial fibrillation (22% vs. 19%; *p*-value = 0.044). Severity of disease, as measured by ICU admission and CURB-65, was not statistically different between vaccinated and non-vaccinated groups. However, a greater percentage of vaccinated patients were in PSI risk class IV–V (63% vs. 54%; *p*-value < 0.001).

### 3.2. Patient Characteristics by PPSV23-Serotype CAP and Non-PPSV23-Serotype CAP Patients

Table 2 depicts patient demographics, social history, and medical history, stratified by PPSV23-serotype CAP and non-PPSV23-serotype CAP patients. The median age for patients with PPSV23-serotype CAP was significantly lower (62 vs. 66; *p*-value = 0.006). A larger percentage of PPSV23-serotype CAP patients were current or former smokers (77% vs. 70%; *p*-value = 0.009) and had a history of non-cirrhotic liver disease (10% vs. 7%; *p*-value = 0.022). The PPSV23-serotype CAP patients had significantly lower rates of heart failure (24% vs. 30%; *p*-value = 0.032), coronary artery disease (22% vs. 30%; *p*-value = 0.002), hyperlipidemia (37% vs. 43%; *p*-value = 0.038) and atrial fibrillation (14% vs. 20%; *p*-value = 0.014). Severity of disease, as measured by PSI risk class IV–V and CURB-65, was not statistically different between PPSV23-serotype CAP and non-PPSV23-serotype CAP patients. However, PPSV23-serotype CAP patients were directly admitted to the ICU at a higher rate (22% vs. 16%; *p*-value = 0.005).

### 3.3. Vaccine Effectiveness of PPSV23 in Overall Cohort

Unadjusted and adjusted estimates of PPSV23 vaccine effectiveness are depicted in Table 3. Overall, PPSV23-serotype CAP was identified in 48 (8%) vaccinated patients and 288 (9%) non-vaccinated patients. The unadjusted effectiveness of PPSV23 vaccination was 17% (95% CI: −13% to 40%). After adjusting for potential covariates, the effectiveness of the PPSV23 vaccine for the prevention of hospitalized PPSV23-serotype CAP was 14% (95% CI: −17% to 39%).

### 3.4. Subgroup Analyses: Vaccine Effectiveness of PPSV23

Unadjusted and adjusted vaccine effectiveness of the PPSV23 effectiveness for the prevention of PPSV23-serotype CAP in six subgroups of interest are depicted in Table 4. Among 1922 patients aged 65 and older, PPSV23-serotype CAP was identified in 27 (8%) PPSV23-vaccinated patients and in 117 (7%) non-vaccinated patients. The unadjusted effectiveness of PPSV23 vaccination was −1% (95% CI: −54% to 36%). After adjusting for potential covariates, the effectiveness of the PPSV23 was 2% (95% CI: −50% to 38%).

A total of 614 patients were admitted directly to the ICU, in which PPSV23-serotype CAP was identified in 9 (9%) PPSV23-vaccinated patients and in 66 (13%) non-vaccinated patients. The unadjusted effectiveness of PPSV23 vaccination was 35% (95% CI: −28% to 71%), and the adjusted vaccine effectiveness was 36% (95% CI: −27% to 71%). Among 3072 patients admitted directly to the ward, PPSV23-serotype CAP was identified in 39 (9%) PPSV23-vaccinated patients and in 222 (9%) non-vaccinated patients. The unadjusted effectiveness of the PPSV23 vaccination was 12% (95% CI: −25% to 39%), and the adjusted vaccine effectiveness was 7% (95% CI: −31% to 36%).

In 2057 patients in PSI risk classes IV–V, PPSV23-serotype CAP was identified in 29 (8%) PPSV23-vaccinated patients and in 160 (10%) non-vaccinated patients. The unadjusted effectiveness of PPSV23 vaccination was 22% (95% CI: −16% to 49%), and the adjusted vaccine effectiveness was 21% (95% CI: −19% to 49%). For 1629 patients in PSI risk classes I–III, PPSV23-serotype CAP was identified in 19 (8%) PPSV23-vaccinated patients and in 128 (9%) non-vaccinated patients. The unadjusted effectiveness of PPSV23 vaccination was 9% (95% CI: −47% to 47%), and the adjusted vaccine effectiveness was 8% (95% CI: −50% to 46%).

For the 351 patients with CURB-65 scores of 4 or 5, PPSV23-serotype CAP was identified in two (3%) PPSV23-vaccinated patients and in 24 (8%) non-vaccinated patients. The unadjusted effectiveness of PPSV23 vaccination was 64% (95% CI: −26% to 94%), and the adjusted vaccine effectiveness was 64% (95% CI: −30% to 94%). For 3335 patients with CURB-65 scores of 0–3, PPSV23-serotype CAP was identified in 46 (8%) PPSV23-vaccinated patients and in 264 (9%) non-vaccinated patients. The unadjusted effectiveness of PPSV23 vaccination was 12% (95% CI: −21% to 37%), and the adjusted vaccine effectiveness was 9% (95% CI: −26% to 35%).

### 3.5. Sensitivity Analysis

Results from the sensitivity analysis were similar to those for the analysis performed on the overall cohort. After excluding 61 patients who received a dose of the PPSV23 in the 30 days prior to, or more than five years prior to their CAP admission, the unadjusted effectiveness of the PPSV23 was 17% (95% CI: −13% to 40%). After adjusting for potential cofounders, the effectiveness was 14% (95% CI: −17% to 38%).

## 4. Discussion

Our study shows that PPSV23 vaccination does not prevent hospitalization due to pneumococcal CAP from vaccine-type serotypes. This is the first study from the United States to estimate the effectiveness of the PPSV23 using urinary antigen detection as the main assay for serotyping.

In our study, we found that PPSV23 vaccination does prevent hospitalization due to vaccine-type CAP in the overall cohort and in each subgroup. In the United States, PPSV23 vaccination is indicated for use in individuals aged 65 and older, however, we found that PPSV23 provided no benefit for hospitalization due to vaccine-type CAP. The same lack of protection was demonstrated among patients admitted to the ICU with PPSV23-serotype CAP, and in patients with a higher PSI risk class and CURB-65 scores.

Current vaccination guidelines suggest a booster dose of the PPSV23 vaccine 5 years after the initial dose [11]. Because of this, our sensitivity analysis excluded patients who received their PPSV23 vaccination more than 5 years before their hospitalization for CAP. We found no difference in removing these patients versus treating them as non-vaccinated in our analysis.

Two studies have reported PPSV23-specific estimates for the prevention of PPSV23-serotype CAP, in which urinary antigen detection for *S. pneumoniae* serotyping was used [14,15]. Lawrence et al. concluded that PPSV23 vaccination was 24% effective (95% CI: 5% to 40%) in preventing hospitalization due to PPSV23-serotype CAP in patients vaccinated with the 15 years prior to hospitalization [14]. Additionally, Lawrence et al. reported an effectiveness of −7% (95% CI: −54% to 26%) for those vaccinated within 0–5 years prior to hospitalization when compared to non-vaccinated individuals. This data is in accordance with our findings. In a study of patients aged 65 and older, Heo et al. found that PPSV23 vaccination did not provide protection against PPSV23-serotype CAP, with an adjusted vaccine effectiveness of 6.3% (95% CI: −73.8% to 49.5%) [15]. This data is in accordance with our subgroup findings.

Two additional studies have reported PPSV23 serotype-specific estimates; however, serotyping in these studies relied on culture-based techniques of blood or sputum. Kim et al. reported a PPSV23 effectiveness of 90.6% (95% CI: 27.6% to 98.8%) against PPSV23-serotype invasive-pneumococcal disease among individuals aged 65–74 in the Republic of Korea [5]. The authors also concluded that PPSV23 vaccination did not prevent non-bacteremic PPSV23-serotype pneumococcal pneumonia in patients aged 65 and older. These findings echo data published by the CDC, which reports that PPSV23 vaccination provides protection against invasive pneumococcal disease [6]. In Japan, Suzuki et al. found that PPSV23 vaccination was 33.5% effective in preventing PPSV23-serotype pneumococcal pneumonia in people aged 65 and older by serotyping sputum [3].

An additional study from Japan among adults ages 65 and older with chronic respiratory diseases by Mesuda et al. found that the odds of all-cause pneumococcal pneumonia were lower in PPSV23-vaccinated patients. The authors also reported a protective effect in a subgroup of patients ages 70 and older [4]. Although these findings do not align with our study, Mesuda et al. did not quantify serotype-specific estimates. Another study among elderly adults in Spain by Domínguez et al. reported a modest trend towards the prevention of CAP hospitalization, ICU admission, and death in PPSV23 vaccinated individuals. However, the findings from Domínguez et al. were not of statistical significance [16]. Similarly to Mesuda et al., Domínguez et al. did not report serotype-specific estimates.

We acknowledge two main limitations in our study. First, urine samples and vaccination records were not obtainable in all hospitalizations. This resulted in 5952 excluded patients, which may affect the external validity of our results. Second, our study is limited to patients hospitalized due to CAP. We cannot assess the effectiveness of the PPSV23 for the prevention of non-hospitalized CAP due to PPSV23 serotypes.

The main strength of our study is the detection of *S. pneumoniae* serotypes through urinary antigen testing. The UAD method was clinically validated and is a powerful tool in assessing the efficacy of pneumococcal vaccines [8]. Additionally, we believe that PPSV23 vaccination was assessed fairly because we only accounted for vaccinations in patients who had an adequate amount of time to develop and maintain antibody levels. The CDC previously reported that 80% of healthy adults will develop antibodies 2 to 3 weeks after PPSV23 vaccination, with elevated antibody levels persisting for more than 5 years [6].

Another strength of our study is that we did not rely on patient recall for proof of vaccination. All proof-of-vaccination data in our study was obtained from medical records or insurance companies. Previously, we demonstrated that proof of vaccination, when obtained from patient recall, is unreliable in adults. We found that subjective documentation of vaccination history was inaccurate in 1023 (37%) of 2787 patients [17].

## 5. Conclusions

Our findings suggest that vaccination with the PPSV23 fails to offer protection against hospitalization due to PPSV23-serotype CAP. This study highlights the need for more effective vaccines in the prevention of hospitalization due to *S. pneumoniae* CAP.

## Figures and Tables

**Table 1:** Patient demographics, social and medical history between PPSV23-vaccinated and non-vaccinated CAP patients.

Variable	PPSV23-Vaccinated (*n* = 608)	Non-Vaccinated (*n* = 3078)	*p*-Value
Age, median (IQR)	68 (59, 76)	65 (53, 77)	0.001
Male sex, *n* (%)	301 (50)	1461 (47)	0.381
Black or African American race, *n* (%)	114 (19)	622 (20)	0.444
Nursing home resident, *n* (%)	59 (10)	256 (8)	0.299
Current or former smoker, *n* (%)	450 (74)	2170 (71)	0.090
Obesity (BMI > 30), *n* (%)	235 (39)	1170 (38)	0.815
COPD, *n* (%)	350 (58)	1442 (47)	<0.001
Diabetes, *n* (%)	227 (37)	969 (31)	0.006
Renal disease, *n* (%)	198 (33)	787 (26)	<0.001
Heart failure, *n* (%)	218 (36)	852 (28)	<0.001
Coronary artery disease, *n* (%)	223 (37)	867 (28)	<0.001
Cerebrovascular disease, *n* (%)	78 (13)	373 (12)	0.674
Neoplastic disease (active or within past year), *n* (%)	80 (13)	405 (13)	>0.999
Liver disease (non-cirrhotic), *n* (%)	53 (9)	212 (7)	0.131
Cirrhosis, *n* (%)	9 (1)	48 (2)	>0.999
Essential arterial hypertension, *n* (%)	469 (77)	2047 (67)	<0.001
Hyperlipidemia, *n* (%)	324 (53)	1250 (41)	<0.001
Prior myocardial infarction, *n* (%)	108 (18)	377 (12)	<0.001
Atrial fibrillation, *n* (%)	135 (22)	572 (19)	0.044
HIV, *n* (%)	8 (1)	52 (2)	0.624
Direct ICU admission, *n* (%)	103 (17)	511 (17)	0.884
PSI risk class IV–V, *n* (%)	381 (63)	1676 (54)	<0.001
CURB-65 score 4 or 5, *n* (%)	63 (10)	288 (9)	0.486

Abbreviations: PPSV23: 23-valent pneumococcal polysaccharide vaccine, IQR: Interquartile range, COPD: Chronic obstructive pulmonary disease, HIV: Human immunodeficiency virus, ICU: Intensive care unit, PSI: Pneumonia Severity Index.

**Table 2:** Patient demographics, social and medical history between PPSV23-serotype CAP and non-PPSV23-serotype CAP patients.

Variable	PPSV23 Serotype CAP (*n* = 336)	Non-PPSV23 Serotype CAP (*n* = 3350)	*p*-Value
Age, median (IQR)	62 (55, 72)	66 (55, 77)	0.006
Male sex, *n* (%)	156 (46)	1606 (48)	0.637
Black or African American race, *n* (%)	60 (18)	676 (20)	0.345
Nursing home resident, *n* (%)	23 (7)	292 (9)	0.286
Current or former smoker, *n* (%)	260 (77)	2360 (70)	0.009
Obesity (BMI > 30), *n* (%)	121 (36)	1284 (38)	0.431
COPD, *n* (%)	180 (54)	1612 (48)	0.064
Diabetes, *n* (%)	95 (28)	1101 (33)	0.098
Renal disease, *n* (%)	79 (24)	906 (27)	0.183
Heart failure, *n* (%)	80 (24)	990 (30)	0.032
Coronary artery disease, *n* (%)	74 (22)	1016 (30)	0.002
Cerebrovascular disease, *n* (%)	33 (10)	418 (12)	0.184
Neoplastic disease (active or within past year), *n* (%)	38 (11)	447 (13)	0.334
Liver disease (non-cirrhotic), *n* (%)	35 (10)	230 (7)	0.022
Cirrhosis, *n* (%)	9 (3)	48 (1)	0.125
Essential arterial hypertension, *n* (%)	219 (65)	2297 (69)	0.226
Hyperlipidemia, *n* (%)	125 (37)	1449 (43)	0.038
Prior myocardial infarction, *n* (%)	36 (11)	449 (13)	0.192
Atrial fibrillation, *n* (%)	47 (14)	660 (20)	0.014
HIV, *n* (%)	10 (3)	50 (1)	0.068
Direct ICU admission, *n* (%)	75 (22)	539 (16)	0.005
PSI risk class IV–V, *n* (%)	189 (56)	1868 (56)	0.909
CURB-65 score 4 or 5, *n* (%)	26 (8)	325 (10)	0.284

Abbreviations: PPSV23: 23-valent pneumococcal polysaccharide vaccine, IQR: Interquartile range, COPD: Chronic obstructive pulmonary disease, HIV: Human immunodeficiency virus, ICU: Intensive care unit, PSI: Pneumonia Severity Index.

**Table 3:** Unadjusted and covariate-adjusted PPSV23 vaccine effectiveness for the prevention of hospitalized PPSV23-serotype CAP.

	Cases *n* (%)	Controls *n* (%)	Unadjusted VE % (95% CI)	Adjusted VE % (95% CI)	*p*-Value
**Overall Cohort, *n* = 3866**					
Number	336	3350	17% (−13 to 40%)	14% (−17% to 39%)	0.350
PPSV23 vaccinated	48 (8)	560 (92)			
Non-vaccinated	288 (9)	2790 (91)			

PPSV23: Pneumococcal polysaccharide vaccine; VE: Vaccine effectiveness. Cases: Patients hospitalized with CAP with positive urinary antigen detection for any of the 23 *S. pneumoniae* serotypes found in the PPSV23 vaccine. Controls: Patients hospitalized with CAP with negative urinary antigen detection for any of the 23 *S. pneumoniae* serotypes found in the PPSV23 vaccine.

**Table 4:** Unadjusted and covariate-adjusted PPSV23 vaccine effectiveness for the prevention of hospitalized PPSV23-serotype CAP in various subgroups.

	Cases *n* (%)	Controls *n* (%)	Unadjusted VE % (95% CI)	Adjusted VE % (95% CI)	*p*-Value
**Patients aged 65 and older, *n =* 1922**					
Number	144	1778	−1% (−54% to 36%)	2% (−50% to 38%)	0.938
PPSV23 vaccinated	27 (8)	331 (92)			
Non-vaccinated	117 (7)	1447 (93)			
**Admitted directly to the Ward, *n =* 3072**					
Number	261	2811	12% (−25% to 39%)	7% (−31% to 36%)	0.685
PPSV23 vaccinated	39 (9)	466 (92)			
Non-vaccinated	222 (9)	2345 (91)			
**Admitted directly to the ICU, *n =* 614**					
Number	75	539	35% (−28% to 71%)	36% (−27% to 71%)	0.232
PPSV23 vaccinated	9 (9)	94 (91)			
Non-vaccinated	66 (13)	445 (87)			
**PSI Risk Class I–III, *n =* 1629**					
Number	147	1482	9% (−47% to 47%)	8% (−50% to 46%)	0.756
PPSV23 vaccinated	19 (8)	208 (92)			
Non-vaccinated	128 (9)	1274 (91)			
**PSI Risk Class IV–V, *n =* 2057**					
Number	189	1868	22% (−16% to 49%)	21% (−19% to 49%)	0.276
PPSV23 vaccinated	29 (8)	352 (92)			
Non-vaccinated	160 (10)	1516 (90)			
**CURB-65 Score 0–3, *n* = 3335**					
Number	310	3025	12% (−21% to 37%)	9% (−26% to 35%)	0.587
PPSV23 vaccinated	46 (8)	499 (92)			
Non-vaccinated	264 (9)	2526 (91)			
**CURB-65 Score 4–5, *n =* 351**					
Number	26	325	64% (−26% to 94%)	64% (−30% to 94%)	0.182
PPSV23 vaccinated	2 (3)	61 (97)			
Non-vaccinated	24 (8)	264 (91)			

PPSV23: Pneumococcal polysaccharide vaccine, VE: Vaccine effectiveness, ICU: Intensive care unit; PSI: Pneumonia Severity Index, Cases: Patients hospitalized with CAP with positive urinary antigen detection for any of the 23 *S. pneumoniae* serotypes found in the PPSV23 vaccine, Controls: Patients hospitalized with CAP with negative urinary antigen detection for any of the 23 *S. pneumoniae* serotypes found in the PPSV23 vaccine.

## Data Availability

Data is available upon request.
